# Children’s representation of specialized skilled movements: The cases of snowboarding and aikido

**DOI:** 10.3758/s13421-024-01522-x

**Published:** 2024-02-09

**Authors:** Allegra Sosic, Sabrina Panesi, Sergio Morra

**Affiliations:** 1 Dipartimento di Scienze della Formazione, Università di Genova, Corso Andrea Podestà 2, 16128 Genova, Italy; 2 Institute for Educational Technology, National Research Council of Italy, Genova, Italy

**Keywords:** Drawing flexibility, Children’s drawing, Snowboarding, Aikido, Working memory

## Abstract

This article investigates children’s graphic representation of two complex motor skills, snowboarding and aikido, from the perspective of drawing flexibility research. In particular, the role of working memory capacity in the development of drawing flexibility is examined. A total of 127 children in the age range 5.7–11.9 years were shown short videos of snowboarding and aikido and were required to make drawings of them. In addition, participants were administered Goodenough’s Draw-a-man Test (that measures the ability to draw detail and proportion in the human figure) and two working memory tests (the Mr. Cucumber test and the Backward Digit Span). The snowboarding and aikido drawings were scored for 19 or 13 features, respectively, on which they could differ from the participant’s standard drawing of a person. The snowboarding and aikido scores were correlated, also controlling for age and Draw-a-man scores, indicating a common variance for drawing flexibility. The drawing flexibility scores increased with age, and were correlated with working memory capacity, also controlling for age and Draw-a-man scores. These results are consistent with a neo-Piagetian model of drawing flexibility development. Detailed analyses are also provided on children’s production of stick figures and “transparencies,” and on the relation of each single modified feature with age and working memory capacity.

**Supplementary Information:**

The online version contains supplementary material available at 10.3758/s13421-024-01522-x.

## Introduction

Children’s ability to draw specialized, skilled human movements is definitely under-investigated. This is perhaps surprising, because it is teachers’ and parents’ commonplace experience that children often draw persons doing sports, dancing, or some specific work. Successful depiction of such topics would be a particular case of “drawing flexibility,” which indicates the child’s ability to modify a habitual drawing scheme in order to represent specific features or conditions of the depicted item. Children’s drawings tend to be schematic (Lowenfeld & Brittain, 1975), and, in particular for the human figure, canonical frontal representation of a static person standing upright is most common (e.g., Cox, 1993). Drawing a person engaged in a particular activity (e.g., doing a sport) that involves specialized movements requires the ability to modify, sometimes deeply, the child’s habitual drawing scheme for the human figure.

So far, only few studies have investigated how children draw these topics, with the notable exception of drawing a scientist (e.g., Finson, 2002); however, this line of research focused on stereotypes of scientists, and in particular gender stereotypes (Miller et al., 2018; Steinke et al., 2007), whereas representation of human movement was irrelevant in this case. A few studies required children to draw sports, but rarely did they consider movement representation. For instance, Colley et al. (2005) required 9- to 16-year-olds to draw a person “who does a lot of sports,” but they focused on whether the participants drew male or female figures and famous or not famous people, while reporting little detail about the drawings. MacPhail and Kinchin (2004) and Mowling et al. (2006) asked schoolchildren to draw their experiences in sport education; however, their analyses focused on children’s attitudes towards sport education, and the coding systems used in these studies convey little information on how body movements were represented. Lange-Küttner and Edelstein (1995) had schoolchildren draw themselves with their friends, and examined the effects of gender, social class, and cognitive ability on the likelihood that participants would draw the human figures as static or in movement; however, they did not report details on which movements were depicted and by which graphic devices. Yüksek Usta and Tezel Șahin (2016) asked 4- to 6-year-olds to draw a picture of sport, and their statistical analyses concerned which sports, which environments, and who was depicted; however, the rich selection of drawings reproduced in this article implicitly suggests that even kindergartners have some ability to modify their habitual drawing scheme of the human figure to represent the sport activities familiar to them.

Perhaps the most relevant study in this regard was carried out by Kapsch and Krugel (2004), who described third- and fourth-graders’ drawings of themselves with the dance artist that had just conducted daily sessions with them for 2 weeks. Kapsch and Krugel (2004) considered children’s ability to modify their habitual representation of the human figure, and described several strategies (such as extension or flexion of the limbs, and sometimes an apparent “regression” to stick figures) used by children to express their embodied experience.

Some studies considered simpler, everyday movements, such as walking, running, or picking up a ball from the ground (Goodnow, 1978; Golomb, 1992; Smith, 1993); Goodnow’s (1978) study was indeed seminal in describing graphic strategies through which children represented those simple movements. Morra (2005) followed up Goodnow’s study by devising detailed scales for those drawing tasks and relating them to experimental and cognitive-developmental variables, as explained below in greater detail. It is now timely to investigate more systematically children’s drawing of human skilled movements, using controlled stimuli, a control condition, an analytic coding system for children’s graphic solutions, and also considering some general aspect of cognitive development.

### Human movement depiction and drawing flexibility

A useful framework for studying children’s depiction of skilled human movements is the line of research on “drawing flexibility,” i.e., the child’s ability to purposely modify a habitual drawing scheme. It has long been recognized that schoolchildren’s drawings are often schematic (Luquet, 1927; Lowenfeld & Brittain, 1975; Van Sommers, 1984). Luquet’s (1927) concepts of “intellectual realism” and “internal model”, i.e., that children draw what they know of the depicted items rather than what they see, were dominant in the field for several decades. However, cognitive psychologists pointed out that drawing is a problem-solving task (e.g., Freeman, 1980); children’s knowledge is by far more extensive than they can represent in drawings (Picard & Vinter, 1999), and Van Sommers (1984) demonstrated that children’s graphic schemes are best characterized as visual memories of their previous solutions of representational problems.

A child’s repertoire of graphic schemes changes in time because of acquisition of new schemes, or when the child is no longer satisfied with a certain pictorial solution and faces the problem of finding a better one. However, graphic schemes can also be accommodated to represent particular meanings. This is often called “drawing flexibility.” Research on drawing flexibility was inaugurated by Goodnow (1978), who studied children’s drawings of a person doing simple movements, such as running or picking up something from the ground. This constitutes a graphic problem, because a conventional drawing of a person standing upright does not adequately represent such actions. An example of drawing flexibility would be drawing a person with a bent trunk and the hands close to the ground, to represent the action of picking up something. Goodnow (1978) classified different strategies used by children in these tasks and reported on how their sophistication improved, at least from kindergarten to grade 3.

To date, some studies of drawing flexibility have used Goodnow’s (1978) tasks. Other studies followed up Silk and Thomas’s (1986) investigation of how young children can differentiate an animal from a human figure. Still others considered the representation of social relations (e.g., friendship; Pinto & Bombi, 2008). Finally, a number of studies used tasks introduced by Karmiloff-Smith (1990), such as drawing a man (or a house) that “does not exist.”

### Explanations of drawing flexibility

There is a lively debate on theoretical accounts of drawing flexibility. Karmiloff-Smith (1990) initially suggested that young children follow rigidly motor routines in drawing until they achieve representational redescription, i.e., conscious access to their procedures that enables them to turn to declarative representations amenable to cognitive change. According to this account, pre-schoolers have acquired drawing procedures (i.e., fixed sequences of motor actions that leave marks on paper) that enable them to depict several items, but they cannot access those procedures metacognitively and re-describe them at higher levels of awareness in order to modify them. Therefore, they are bound to inflexible routines that yield stereotyped drawings. However, subsequent research (Barlow et al., 2003; Berti & Freeman, 1997; Spensley & Taylor, 1999; Zhi et al., 1997) criticized that explanation. These studies indicated that pre-schoolers do not follow rigid drawing procedures – consistent with Van Sommers’s (1984) finding that children’s graphic schemes are based on visual representations (which remain fairly stable), not on motor representations (because the order of strokes can vary greatly over repeated drawings). These studies also found that pre-schoolers do show some flexibility in drawing, and falsified several predictions of the representational redescription model, thus disproving Karmiloff-Smith’s hypothesis (see Barlow et al., 2003; Spensley & Taylor, 1999). Alternatively, information processing accounts of drawing flexibility were proposed (e.g., Barlow et al., 2003; Berti & Freeman, 1997; Simpson et al., 2019), sometimes with an emphasis on working memory and/or inhibition, and often also with a role of other processing components. These accounts are not necessarily in competition with one another, because several factors could be involved in drawing flexibility; in particular, working memory and inhibition are probably complementary, the former being involved in holding and manipulating information that is relevant to solve the representational problem, and the latter in resisting the dominance of habitual but inadequate graphic schemes.

In particular, Morra (2005, 2008a) proposed a cognitive account of drawing flexibility framed within a neo-Piagetian theory. Neo-Piagetian theories (e.g., Pascual-Leone, 1987; see also Morra et al., 2008; Pascual-Leone & Johnson, 2021) retain some Piagetian constructs, such as the construct of scheme, but dismiss the notion of stages defined by logical structures; instead, they place a major emphasis on the development of the information processing system, and in particular on the system-wide effects of working memory capacity growth. Pascual-Leone’s theory retains the Piagetian distinction between figurative and operative schemes (Piaget & Inhelder, 1966; see also Feldman, 2000). A figurative scheme is a representation of a state of affairs (e.g., an object, a particular arrangement of objects, a part or a feature of an object); figurative schemes are often organized hierarchically (e.g., the scheme of a bicycle can include constituent schemes for the wheels, the handlebars, the saddle). According to Pascual-Leone and Johnson (2021, p. 153) “When figurative schemes apply, they together generate a mental state (phenomenal representation), which is an intuitive synthesis, or configural experience, bearing meaning […] Operative schemes are mental or external transformations or actions […] A transformation changes a figurative state into another, distinct and possibly different, figurative state.” In the domain of children’s drawing, graphic figurative schemes are long-term memory representations of the visual appearance of satisfactory depictions of various items, and they have a hierarchical structure, whereby the constituent parts of a graphic scheme denote the parts of the item to be drawn; relevant operative schemes include rules for placing graphic schemes in a two-dimensional space and mental operations that manipulate or modify the constituent parts of a higher-order graphic figurative scheme (Morra, 2008a). Domain-general cognitive processes are also involved in children’s drawing, including the attentional resources that are at the core of working memory.

According to Morra’s (2005, 2008a) model, the modification of a habitual graphic (figurative) scheme requires that, in addition to the graphic scheme itself, other schemes are also activated in the child’s mind. These would include figurative representations of features of the specific subject to be depicted (e.g., when drawing a person picking up something from the ground, what specific features of a person characterize this action?) and an operative scheme for modifying components of the habitual graphic scheme in order to depict the intended specific feature(s). This would place a remarkable information load on working memory, especially when a “global” modification of a graphic scheme is involved. For instance, to draw a person picking up something from the ground, a child may want to draw the person with a tilted body axis; in this case, one could not start by drawing an upright head as usual, because a tilted body axis also entails drawing the head in a different position. In general, advance planning is necessary in “global” changes, where altering some part of a drawing also affects how other parts must be drawn, and the relation between a totality and its parts needs to be considered. For such planning, several schemes need to be coordinated, for example, a graphic figurative scheme for the human figure, one or more figurative schemes that represent the visual aspect of a person’s global feature(s) that need to be modified in the drawing, and an operative scheme that mentally transforms those features in the graphic scheme. Therefore, to plan “global” changes, a child must activate several schemes simultaneously, which could place a high demand on working memory. In the case of “local” changes, which involve modifying a single part while performing the drawing, the working memory load would be less heavy. For instance, to draw a person who is running, a child might start drawing as usual the upper half of a human figure, and only then decide to draw the legs spaced farther apart. This involves coordinating two schemes, one figurative (e.g., a mental image of the legs in a running person) and one operative to modify the stereotyped drawing (e.g., widening the angle between the graphic scheme components that represent the legs). However, for 5- or 6-year-olds, even a load of only two schemes could be a bit taxing.

Morra (2005) suggested that the schemes required for drawing flexibility could be activated using three resources. First, endogenous (i.e., mental) attention that is the core of working memory capacity according to current theories, and the capacity of which grows with age (Cowan, 2016; Pascual-Leone & Johnson, 2021; Portrat et al., 2009). In particular, in Pascual-Leone’s theory, the capacity of this attentional resource (also called M capacity) in typical 5-year-olds is limited to activating two schemes, and it grows on average by one unit every second year until adolescence (Pascual-Leone, 1987). Perceptual input is another possible source of activation of relevant schemes. If a model is available while drawing (e.g., a photo of the subject), the child can note some of its relevant features, activate with little effort their mental representations, and try ways to render them in the pictorial execution. Third, contextual elements could prompt executive control (e.g., Davis, 1983); for instance, if a child is required to draw a person who is standing still, and after that a person who is running, the order of these requests may alert the child to make the second drawing look different from the first, and thus to inhibit the tendency to draw the habitual scheme of the human figure, and search mentally for features that could be relevant in a modified drawing.

A series of three experiments (Morra, 2005) provided clear evidence for all three sources of activation. Further research (Blom et al., 2021; Panesi & Morra, 2016, 2022; Simpson et al., 2019) provided additional support for the role of working memory and/or inhibitory control in drawing flexibility with the drawing topics traditionally used in that line of research. The investigation of children’s drawing of human skilled movements can benefit from the extensive previous research on drawing flexibility and, in turn, contribute to extending our knowledge of children’s drawing flexibility.

### The current study

The investigation of children’s drawing of human skilled movements can benefit from previous research on drawing flexibility, because that line of research provides a framework for analyzing the changes made by children with respect to their habitual drawing schemes and the role of at least some underlying cognitive processes. In this article we report on drawings of snowboarding and aikido made by children from kindergarten to grade 5. These drawings were compared with the drawings of a still person made by the same children. To avoid any influence of children’s specific experience, and to control for the content to be represented, we selected two activities (snowboarding and aikido) with which all participants were unfamiliar, and presented them with short videos and a few freeze frames from the same videos.^1^ Snowboarding is a winter sport practiced individually with specific equipment (the board), descending on snow-covered slopes along routes of varying difficulty, sometimes equipped with poles or springboards, and sometimes performing acrobatics. Aikido is a traditional Japanese martial art (not a competitive sport), most often performed in pairs where the partners take turns in the roles of attacker and attacked, and the attacked person performs sophisticated martial techniques that exploit the energy of the attack to throw or bring under control the attacker.

The method of this study follows closely that of Morra (2005); in particular, the participants were tested for working memory capacity with the same tests used by Morra (2005). Thus, we explore children’s graphic representations of two specific high-level motor skills relying on the methods and the developmental approach of drawing flexibility research. The specific hypotheses of this study, listed below, follow consequentially from our analysis of the literature. However, our study was not preregistered; in fact, it is largely exploratory, given that (to the best of our knowledge) it is the first study on drawing either snowboarding or aikido, and also the first study on children’s drawing that uses video stimuli.

This investigation can also contribute to our knowledge of children’s drawing flexibility by extending it to the important but under-investigated topic of specialized, skilled human movements (also using a novel method, i.e., presenting video stimuli). In this article, we shall first describe informally some pictorial strategies used by children of different ages, in order to provide the reader with some global, intuitive understanding of how the participants approached the task and represented the content of the videos, before turning to analytical data on the features of their drawings and statistical hypothesis testing. The following hypotheses and predictions are tested: (a) The flexibility scores in the snowboarding and aikido drawings share a significant portion of variance; (b) drawing flexibility scores increase with age; (c) there could be a gender difference, consistent with some previous findings that highlighted girls’ better ability to represent detail in their drawings (e.g., Lange-Küttner & Ebersbach, 2013); (d) working memory capacity is a major predictor of drawing flexibility scores, which would extend the empirical support for the neo-Piagetian account of drawing flexibility (Morra, 2005, 2008a; Panesi & Morra, 2016) to the domain of skilled human movement representation; (e) working memory capacity growth accounts at least in part for the increase with age of drawing flexibility scores, also consistent with Morra (2005); (f) the pictorial problem of representing specialized skilled movements could induce children to use pictorial devices (perhaps regressive, or perhaps strategic) such as transparencies or stick figures.

## Method

### Participants

A total of 127 typically developing children (64 girls, 63 boys) in the age range 5.63–11.79 years (mean age 8.71, SD = 1.59 years) took part in this study. They were all children from kindergarten to grade 5 who received written parental consent in the schools located in two villages (approximately 2,000 inhabitants each) in Northern Italy. Ethnicity was largely Caucasian (90%), with the rest being Arab (9%) and mixed Caucasian-African (1%). The participants included six Ukrainian refugee children, for whom the instructions were translated and the digits read in the Ukrainian language; they are included in the analyses, because there was no evidence that they performed differently from the rest of the sample. All other participants were either Italian monolinguals or children of immigrants who had been in Italy for several years and spoke Italian fluently; they were all tested in Italian. For some analyses we divided the sample into three age groups – younger (5.63–7.54, mean = 6.65, SD = 0.65 years), middle (7.63–9.54, mean = 8.67, SD = 0.63 years), and older (9.63–11.79, mean = 10.53, SD = 0.61 years).

### Materials and procedure

All participants were tested individually in a quiet room. The drawings were made with a pencil on A4 sheets (one for each drawing). The drawing of a person was required first, with the standard instructions of Goodenough’s (1926/1977) Draw-a-man test (see Online Supplementary Materials (OSM)).

Subsequently, drawings of snowboarding and aikido were made, approximately half of the participants starting with snowboarding and half with aikido. The participants knew in advance that they would need to draw, because before testing the experimenter went to the classrooms to introduce herself and explained to the children that she would show them some interesting videos and then ask them to make drawings of those videos. For the snowboarding drawing, the child was shown on a computer screen three videos lasting 11, 16, and 8 s, respectively, each followed by a freeze frame from that video for 5 s (for a total duration of 50 s); then the experimenter asked: “Would you please make a drawing of what you have seen?” For the aikido drawing, the child was shown two videos lasting 24 and 32 s, respectively, each followed by a freeze frame from that video for 5 s (for a total duration of 66 s); then the experimenter asked: “Would you please make a drawing of what you have seen?” (Note that only one snowboarding drawing was required after viewing all three snowboarding videos and, similarly, only one aikido drawing after both aikido videos.) The reason for using three shorter videos for snowboarding and two slightly longer ones for aikido is that the speed of movement is much faster in snowboarding than in aikido. We selected videos that, according to our own experience with these activities, could be sufficiently rich in information but not too long or overwhelmingly detailed. Each aikido video showed highly experienced *aikidoka* performing throw (*nage*) techniques; the protagonists were women in one video and men in the other. Each snowboarding video showed an experienced snowboarder (whose gender was not really detectable because of the outfit, which included a wind jacket and a helmet) descending a slope and performing some acrobatics with springboards or rails. The freeze frames were created with the purpose of highlighting a salient moment of each video, in which some important features of the protagonist’s action were clearly visible; each freeze frame was presented for only 5 s, assuming that this duration would be sufficient to enhance attention to some features of a movement, but not for thoroughly studying the image and using it as a model to copy. The stimulus videos and freeze frames can be retrieved from https://drive.google.com/drive/folders/1nJS-zksyZfucivTusvA3x6PvA4VFgVIu?usp=sharing and are included as Online Supplementary Materials (OSM) to this article.

Finally, working memory capacity was assessed with the Mr. Cucumber Test and the Backward Digit Span. These are the same working memory tests used by Morra (2005). They are consistent with the neo-Piagetian framework of this study (e.g., see Case, 1985), they have different content, and they are suitable for the whole age range of our participants. By averaging the scores in both tests, one can estimate the capacity of a central attentional resource that is at the core of working memory (see Morra, 1994); this domain-general attentional resource is called M capacity in some neo-Piagetian theories, which also define its scale of measurement (e.g., see Pascual-Leone & Johnson, 2021). The Mr. Cucumber test presented outline drawings of an extraterrestrial figure with colored stickers (from one to eight) attached to it; after each colored figure the child was shown a colorless outline and asked to indicate the stickers’ positions. There were three items per level (defined by the number of stickers), in ascending order. Stimuli of levels 1–5 were presented for 5 s; more complex stimuli were presented for as many seconds as the number of colored spots they contained. The test was discontinued when the child failed all items of a given level. The Backward Digit Span included three lists for each length, from two digits up to eight. The experimenter read aloud the digits at a rate of approximately 1.5 s per digit. The test was discontinued when the child failed all three lists of a given length.

### Scoring

The drawing of a person was scored according to the Goodenough test manual, Italian edition (Goodenough, 1926/1977). The score is based on the presence of different parts and details, their dimensions, and their proportions (see OSM for the full scoring checklist). Moreover, it was used as a control condition for the snowboarding and aikido drawings.

For the snowboarding drawing we prepared a list of 19 features (see Table 1) on which it could differ from the control drawing; for the aikido drawing, we prepared another list of 13 features (see Table 2). We only included in these lists features that belong to the depicted persons or their equipment and outfit, and are relevant for representing the intended activity or movements. These lists were based on the same principles as in previous research (Morra, 2005; Panesi & Morra, 2016). However, we compiled the feature lists only after collecting the participants’ drawings and exploring the graphic devices they used; given the lack of previous research on these specific topics, defining in advance the lists of relevant graphic features would have been unwise. All three authors examined the whole set of drawings and, upon discussion, easily agreed on two lists of features that were relevant according to the aforementioned criteria. Each drawing was then scored 1 or 0 on each feature by the first two authors independently; the third author decided on all cases of disagreement. Then, drawing flexibility scales were created for the snowboarding and aikido drawings summing the scores on the relevant 19 or 13 features, respectively. Detailed examples of scoring are provided in OSM Table S1.

**Table 1:** Features scored in the snowboarding drawing

Label	Description
Tilted board	The board is tilted in a perceivable way with respect to the lower edge of the paper sheet (including 180° rotation when a flip is represented)
Tilted body axis	The body axis is not vertical and is tilted in a perceivable way with respect to the board
Board on slope	The board is drawn along the mountain slope (explicitly represented)
Bent knee	One or both knees bent (also in case they are bent in an unnatural way)
Tilted trunk	The trunk is tilted with respect to the legs or curved, different from the control drawing
Tilted head	The head is tilted with respect to the trunk (with a clear tilt, not simply a difficulty in drawing the neck), different from the control drawing
Jump	The board is clearly detached from the ground or the springboard (explicitly represented)
Flip	The board is in flight, the person’s feet are on the board, and the orientation of the person and board clearly represents a flip
Crouch	Crouching or curled posture; the legs/body height proportion is clearly smaller than in the control drawing
Grab	Hands grabbing the board, i.e., one or both hands in contact with the board
Legs wide apart	Legs clearly wider apart than in the control drawing, or forming an angle while in the control drawing they are parallel
Feet	The feet are more salient (e.g., more elaborated or clearly larger) than in the control drawing and they both are drawn within the board
Angle of feet	Feet on the board, with space between them, and making an angle. The angle is visible as such or represented by foreshortening one foot. Not simply feet pointing outside as in the control drawing
Bindings	Two bindings on the board, in which the feet are inserted
Arms dynamic	Arms in a dynamic posture (e.g., upward or in a sort of momentum or clearly representing an action), different from the control drawing
Arms different	Arms in different postures, and different from the control drawing
Bent elbow	One or both elbows bent (also in case they are bent in an unnatural way). Accept also elbow representations as described in the Goodenough DAM manual, provided they are absent in the control drawing
Outfit	Any relevant piece of clothing/equipment, e.g., helmet, wool beanie, sunglasses, mask, gloves, mittens, or scarf. Score leniently, but it must be different from the control drawing
Lines of movement	Straight lines out of the figure that represent the direction of a fast movement (as sometimes used in comics)

**Table 2:** Features scored in the aikido drawing

Label	Description
Profile	At least one figure in profile (accept also partially successful depictions, e.g., profile head and feet, or profile body and feet), different from the control drawing
Tilted trunk	The trunk is tilted with respect to the legs or curved, different from the control drawing. Can be either tilted backward (a person starting to fall) or a (misconceived) representation of a person tilted forward to perform an attack or a technique
Fall	Any representation of *uke* (the person who receives a technique) falling or already fallen on the ground
Fall details	Details of the fall, e.g., legs upward and at an angle with the body, or a bent leg, or one arm upward and the other making an acute angle with the body. Not just *uke* horizontal with arms making a cross, or in the same posture as in the control drawing although rotated 90°
Step forward	One person upright, with feet in profile and one leg clearly advanced or bent. It can be either *uke* stepping forward to attack, or *seme* (the person who performs a technique – also called *shite* or *tori*) stepping forward while performing a technique
Body contact	Contact or crossing of *seme*’s and *uke*’s bodies
Back foot	The back foot makes an angle with the front foot. The angle is visible as such or represented by foreshortening one foot. Not simply feet pointing outside as in the control drawing
Arm protrusion	One or both arms extended towards the partner. Not two arms wide and symmetrical in a frontal figure
Hand contact	One or both hands making contact with hand(s) or wrist(s) of the partner
Arms dynamic	Arms in a dynamic posture (e.g., upward or in a sort of momentum or clearly representing an action), different from the control drawing
Arms different	The two arms in different postures, and different from the control drawing
Outfit	A recognizable representation of *hakama* or *dogi* or *obi* (accept also a type of *obi* used in other martial arts). Score leniently, but it must be different from the control drawing, and not a typical representation of a short skirt
Lines of movement	Straight lines out of the figure that represent the direction of a fast movement (as sometimes used in comics)

Each drawing was also scored for the presence or absence of transparencies (i.e., cross-over of the contours of two items, so that one of them seems visible through the other) and for the presence or absence of stick figure features (it was not required that the whole person was a stick figure; it was sufficient that the arms or the legs were unidimensional).

For the Mr. Cucumber Test and the Backward Digit Span, one point was given for each consecutive level on which a child was correct on at least two items out of three, plus one-third of a point for each correct item beyond that level. (To make the scores in the two tests comparable, for the Backward Digit Span one point was awarded by default for list length one, because testing started from lists of two digits.)

## Description of selected drawings

Children seemed highly motivated to perform our tasks. To provide the readers with an intuitive comprehension of the outcome, in this section we describe and illustrate some aspects of the observed representational strategies.

Most participants placed the figures in a central area of the paper, and occupied a relatively large part of it, often also depicting contextual elements (e.g., mountain landscapes, other people in the background). Occasionally we observed the use of stick figures to schematically represent a movement (like Kapsch & Krugel, 2004) and an increased use of “transparencies” (e.g., the body or the limbs visible through the outfit). Transparencies and unidimensional representations of arms or legs decreased with age (see the *Results* section for details). In a very few cases, participants assimilated the scenes in the videos with other previous knowledge, so that ski sticks appeared in two snowboarding drawings, and karate postures and outfits in some aikido drawings, but these misconceptions were rare. Especially in the younger group, some drawings seemed focused on the dynamics of the events and the context (at the cost of accurate representation of human figures), while others were focused on the details of the figures (sometimes including details of little relevance, such as the hairstyle of the persons in the videos) that, however, appeared rather static. It seemed difficult for some participants, particularly the youngest ones, to represent the events and the human movements while also caring for the quality of the figures.

Spatial organization broadly followed the trend described by Dennis (1992), from figures “floating” in an unorganized space (although in the snowboarding drawing young children also often included landscapes and contextual elements), to a use of alignments with or without explicit groundlines, to a more systematic use of the two-dimensional graphic space.

In aikido drawings at least two persons were represented, with different nuances of the relationship between them. In some drawings, both figures were standing at a distance (most often as preparing to start an action, but in a few cases, even as if there were no relationship between them). Several participants depicted the outcome of a martial technique, with a person falling or already fallen to the ground. Most participants represented both figures standing and close enough, some of them emphasizing an aggressive interaction, others depicting the persons seemingly holding hands with smiling faces. Aikido is often regarded as a martial art of peace; it seems that some children were more impressed by the martial aspects and others by the peaceful and friendly aspects in the videos. Disregarding these more expressive qualitative features of aikido drawings, we classified them in four categories; 40 participants (31.5%) drew both protagonists standing at a distance, 28 (22.0%) represented both protagonists standing and an action in progress, 47 (37.0%) drew *seme* performing a technique and *uke* 2 falling, and 12 (9.5%) portrayed *seme* standing after having completed an action and *uke* on the ground. This category distribution was unrelated to age.

The fact that aikido drawings necessitated depicting two persons could perhaps challenge the participants with a more complex task than the snowboarding drawings, where only one human figure was needed. On the other hand, drawing two persons could also offer more opportunities for flexibility, because a participant could modify either figure to represent the martial technique being performed or its consequence (the fall of one person).

**Fig. 1:**
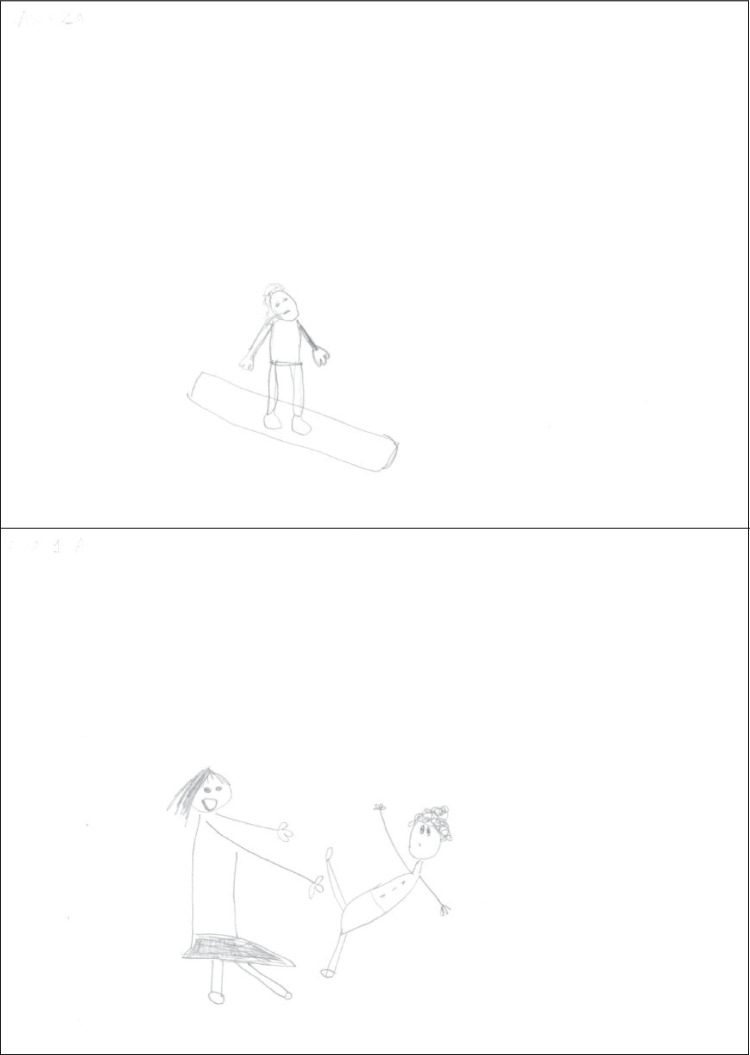
Drawings by a girl aged 6.21 years (scores: snowboarding 2, aikido 6, WM capacity 2.17)

The drawings presented here are not necessarily the most typical at each age, but serve the purpose of illustrating the aforementioned patterns and strategies. Figure 1 shows a kindergartner’s drawings. In both drawings the figures are placed in an empty space. In the snowboarding drawing a transparency is present (board edge visible through the legs). Tilted board and tilted head were scored points. In the aikido drawing the scene appears more dynamic; *seme* has a triumphant smile and *uke* is clearly unbalanced backward, but their arms are reduced to unidimensional lines. Points were scored for step forward, arm protrusion, arms different (in *seme*) and tilted trunk, fall, and fall details (in *uke*).

**Fig. 2:**
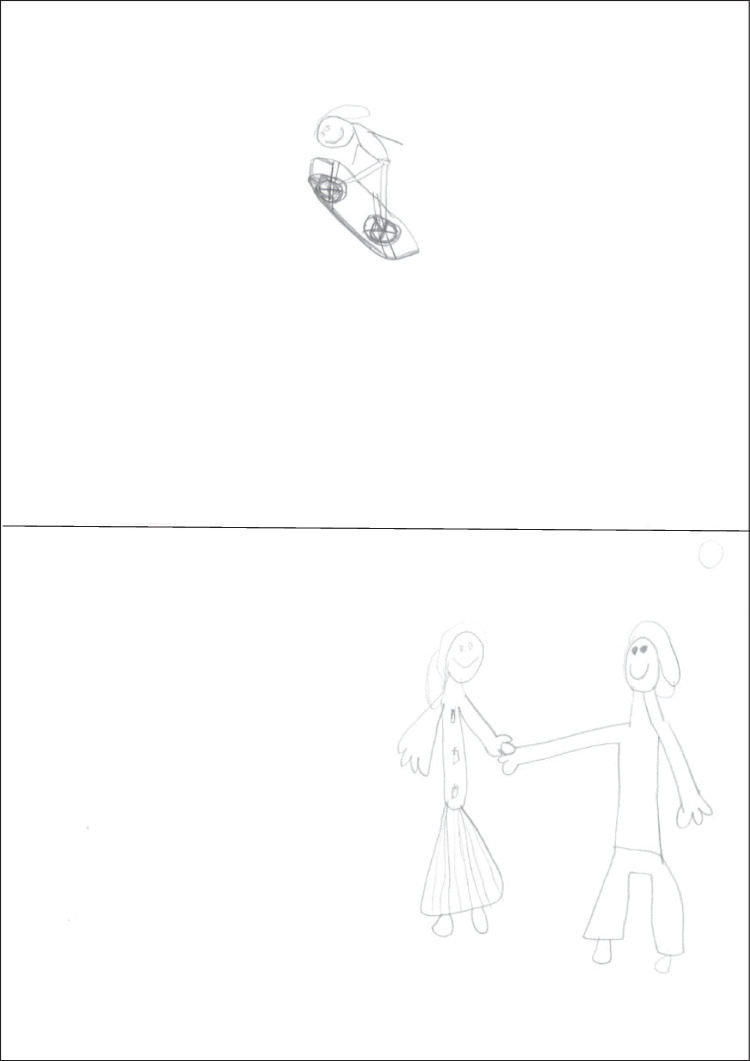
Drawings by a girl aged 7.46 years (scores: snowboarding 6, aikido 4, WM capacity 2.33)

Figure 2 presents a first-grader’s drawings. Here, too, the figures are placed in empty space; the posture in the snowboarding drawing suggests a jump, but this feature was not given a point because no context was represented. The global appearance is rather dynamic, but the figure is impoverished, with unidimensional arms. Points were scored for tilted board, tilted body axis, tilted trunk, tilted head, feet, and bindings. The aikido drawing belongs to the more peaceful type; it is unclear who is attacking. The figures are more elaborate but rather static. Points were scored for arm protrusion, hand contact, arms different, and outfit.

**Fig. 3:**
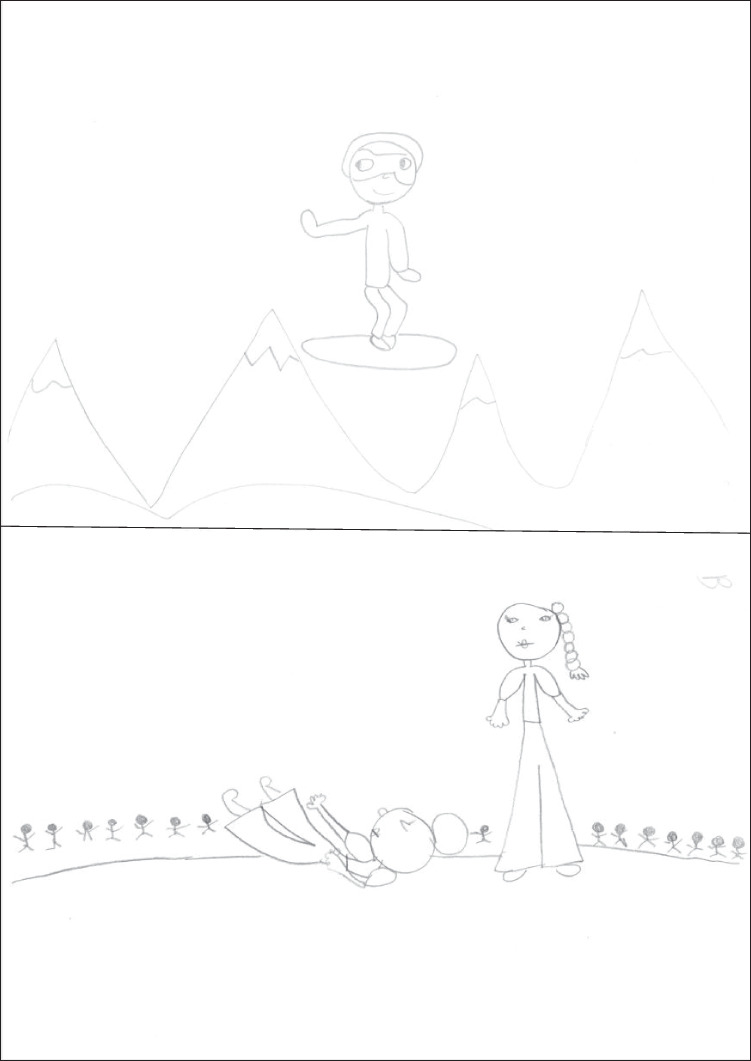
Drawings by a boy aged 7.79 years (scores: snowboarding 5, aikido 6, WM capacity 3.33)

Figure 3 presents a second-grader’s drawings. The spatial context is well organized: a wavy groundline and sharp peaks in the background for snowboard, a horizontal line indicating the end of the ground and schematized people in the background for aikido. Partial occlusions are carefully depicted. The snowboarder figure is quite articulated, with bent knees, dynamic and different arms, and some outfit, but the board is unnaturally horizontal, despite a high jump. The aikido drawing portrays the outcome of a technique: *seme* is now static, but the dynamics of the scene are conveyed by the details of *uke*’s fall.

**Fig. 4:**
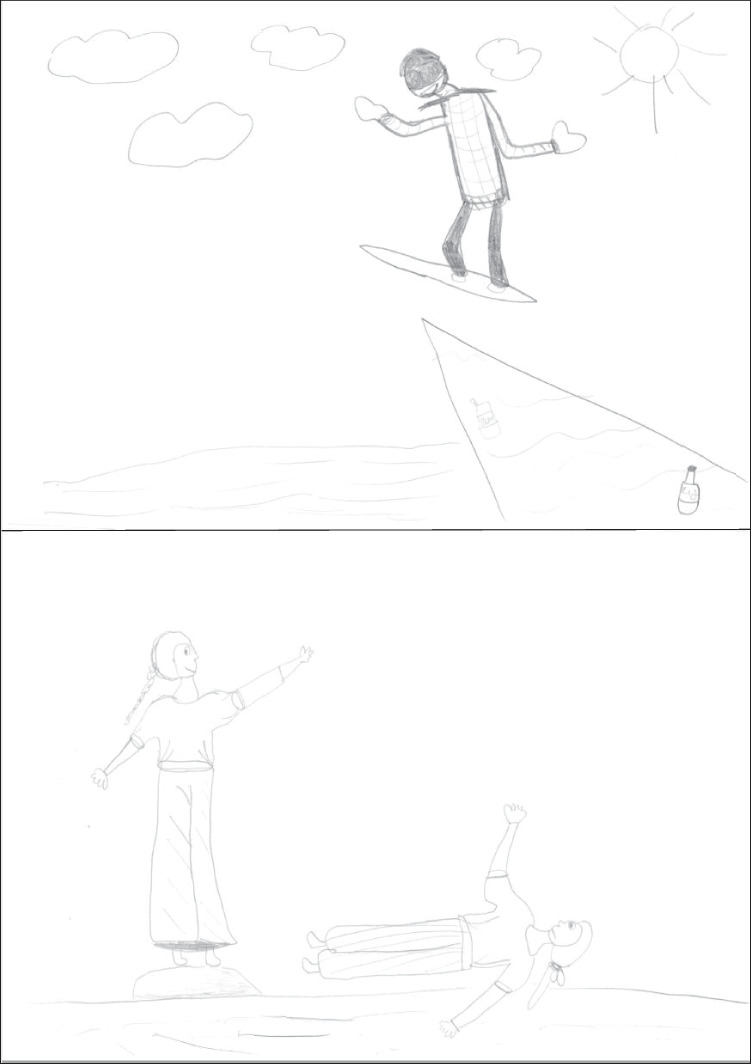
Drawings of a girl aged 8.71 years (scores: snowboarding 11, aikido 7, WM capacity 5.50)

Figure 4 presents a third-grader’s drawings. Contextual elements are minimal but sufficient to provide a spatial structure; a springboard is present in the snowboarding drawing. The snowboarder figure is rich in modified details, particularly in the arms, the legs, and the tilt of various body parts. The aikido drawing shows the conclusion of a technique, with *seme* in action (profile, arm protrusion, arms different, and arm dynamic were scored points) and *uke* on the ground.

**Fig. 5:**
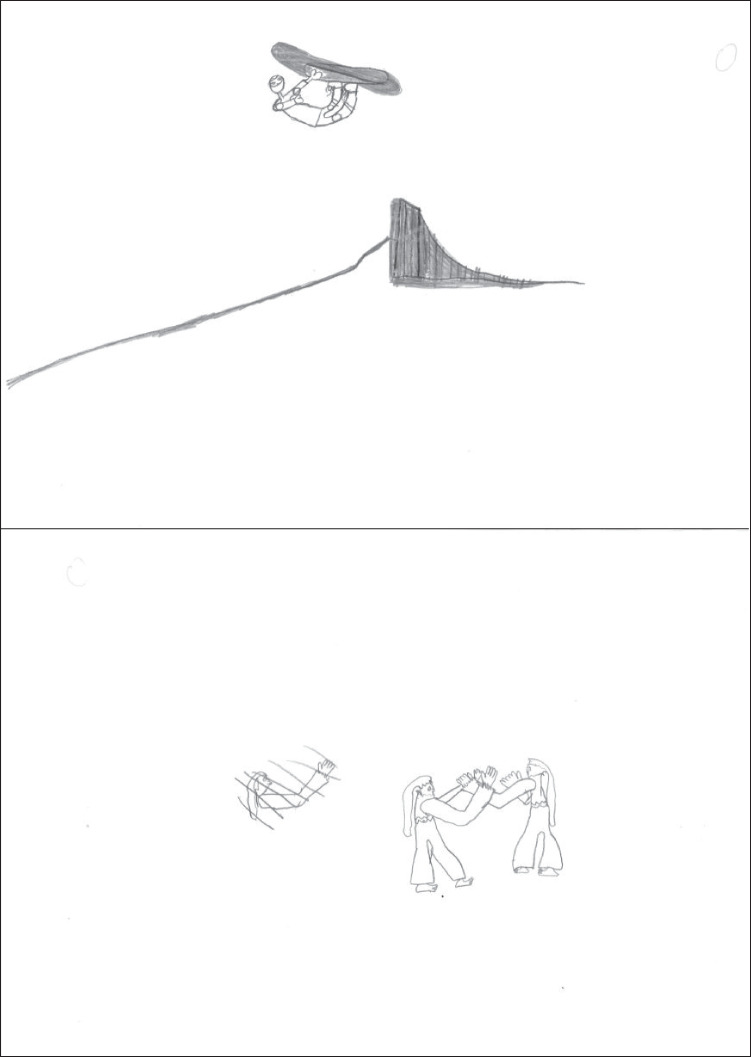
Drawings of a boy aged 9.46 years (scores: snowboarding 12, aikido 7, WM capacity 4.83)

Figure 5 presents a fourth-grader’s drawings. The snowboarding drawing is a remarkable representation of a flip, rich in modified details in the arms, the legs, and various tilted body parts. Only in the snowboarding drawing is some context present. The aikido drawing emphasizes depiction of the arms; both figures are in profile, and *uke* is represented stepping forward and extending both arms to grab *seme*’s left wrist (while *seme*’s right arm is kept in a different posture). This complex pattern of both protagonists’ arms is drawn without any transparency.

**Fig. 6:**
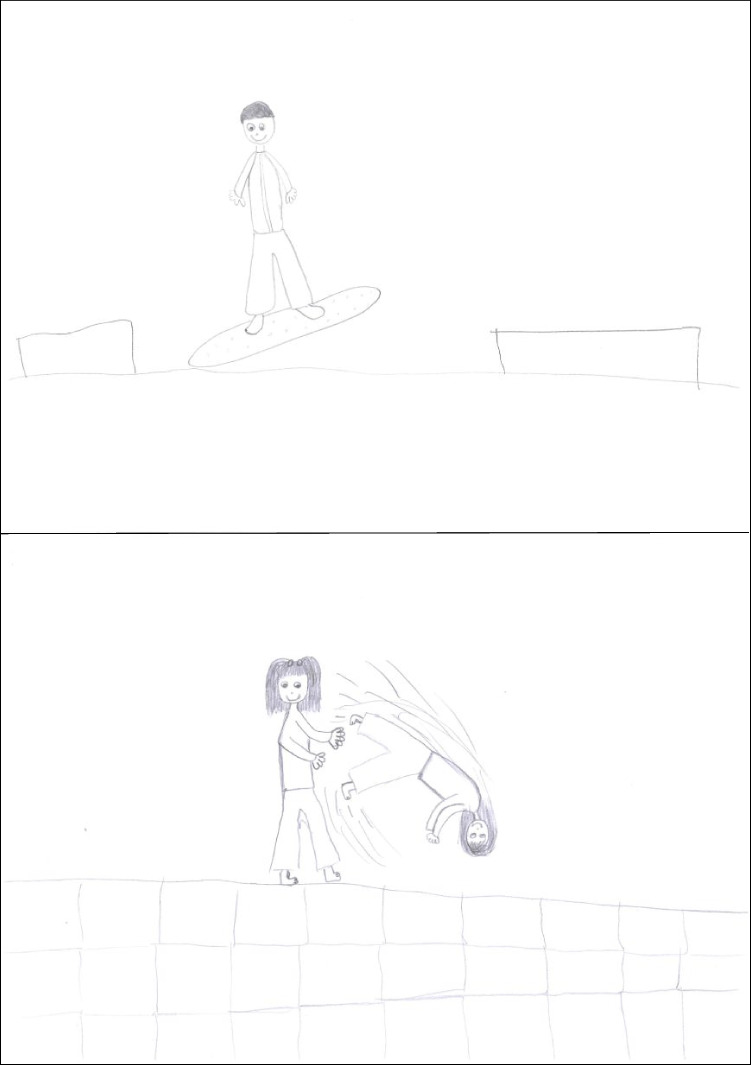
Drawings of a boy aged 11.13 years (scores: snowboarding 3, aikido 6, WM capacity 3.83)

Figure 6 presents a fifth-grader’s drawings. The snowboarding drawing shows little flexibility (only the tilted board, the angle of feet, and the outfit were scored points), and the context consists of a horizontal groundline and two snowboarding rails. The aikido drawing is a remarkable representation of a forward fall, in which the dynamic representation of *uke*’s whole body and arms is accompanied by lines of movement.

Having provided examples of children’s drawings and descriptions of different kinds of representations, we can now turn to more formal data analysis and hypothesis testing.

## Results

### Preliminary analyses

The inter-rater agreement on all snowboarding features was 85.3% and the inter-rater correlation was *r* = .86; Cronbach’s alpha on 19 items was .80. The inter-rater agreement on all aikido features was 86.1% and the inter-rater correlation was *r* = .78; Cronbach’s alpha on 13 items was .68. Cronbach’s alpha is an index of internal consistency of a scale; the lower alpha for the aikido drawing can be explained not only by fewer items, but more importantly by the fact that, because of the different strategies used by participants to represent aikido, some features are necessarily negatively associated (e.g., if *uke* is depicted falling, it is not possible to also have body or hand contact in the same drawing), which may limit the degree of internal consistency of this scale. In sum, reliability (in terms of both inter-rater agreement and scale internal consistency) was good for the snowboarding scale and acceptable for the aikido scale. For a total scale of drawing flexibility, i.e., the sum of the snowboarding and aikido scores (32 items), Cronbach’s alpha was .83.

The mean scores of the Mr. Cucumber Test (3.47) and the Backward Digit Span (3.59) were not significantly different, *t*(126) = 1.71, and the correlation was *r*(125) = .73, *p* < .001; therefore, we averaged them into a single working memory capacity measure.

Table 3 reports the descriptive statistics for the main variables in this study. The values of skewness and kurtosis clearly indicate that all variables well approximated normal distributions, with no floor or ceiling effects.

**Table 3:** Descriptive statistics in the total sample

	Mean	SD	Min	Max	Skew	Kurtosis
Draw-a-man	23.27	5.28	12	35	.06	-.49
Snowboarding scale	5.80	3.77	0	15	.40	-.64
Aikido scale	4.01	2.48	0	10	.12	-.92
Total drawing flexibility	9.81	5.35	0	21	.12	-.76
Snowboarding % scale	30.50	19.86	0	78.95	.40	-.64
Aikido % scale	30.83	19.08	0	76.92	.12	-.92
Average percentage	30.67	16.53	0	64.98	.05	-.84
Mr. Cucumber Test	3.47	1.09	1.00	6.00	.28	-.32
Backward Digit Span	3.59	.95	2.00	6.33	.46	.09
WM capacity (averaged)	3.53	.95	1.50	6.17	.30	-.14

Snowboarding scale and Aikido scale refer to raw scores (number of features scored)

Total drawing flexibility is the sum of Snowboarding scale and the Aikido scale

Snowboarding % scale and Aikido % scale are the percentages of the raw scores over 19 and 13 features, respectively

Average percentage is the average of Snowboarding % and Aikido %

WM capacity (averaged) is the average of Mr. Cucumber Test and Backward Digit Span

Tables 4 and 5 report the proportion of features scored 1 (by age group and in the total sample) in the snowboarding and the aikido drawings, respectively.

**Table 4:** Proportion of 1 scores on each feature of the snowboarding drawing, by age group and in the total sample

	Younger	Middle	Older	Total
Tilted board	.35	.53	.69	.54
Tilted body axis	.16	.32	.33	.28
Board on slope	.10	.11	.10	.10
Bent knee	.10	.30	.38	.28
Tilted trunk	.19	.28	.33	.28
Tilted head	.23	.26	.23	.24
Jump	.26	.47	.54	.44
Flip	.10	.09	.13	.10
Crouch	.03	.11	.21	.12
Grab	.06	.16	.26	.17
Legs wide apart	.32	.39	.46	.39
Feet	.32	.40	.31	.35
Angle of feet	.26	.28	.44	.32
Bindings	.19	.23	.13	.19
Arms dynamic	.35	.44	.59	.46
Arms different	.26	.33	.46	.35
Bent elbow	.03	.12	.26	.14
Outfit	.77	.82	.92	.84
Lines of movement	.19	.21	.15	.19

**Table 5:** Proportion of 1 scores on each feature of the aikido drawing, by age group and in the total sample

	Younger	Middle	Older	Total
Profile	.29	.39	.23	.31
Tilted trunk	.23	.11	.18	.16
Fall	.23	.47	.44	.40
Fall details	.13	.32	.28	.26
Step forward	.16	.21	.28	.22
Body contact	.00	.02	.05	.02
Back foot	.13	.18	.08	.13
Arm protrusion	.45	.61	.64	.58
Hand contact	.16	.14	.13	.14
Arms dynamic	.42	.56	.64	.55
Arms different	.35	.42	.59	.46
Outfit	.52	.68	.77	.67
Lines of movement	.03	.12	.10	.09

### Main analyses

The raw scores of the snowboarding and aikido scales are not directly comparable, because they are based on checklists of 19 and 13 features, respectively; therefore, to make them comparable, we converted them to percentages of the maximum possible score. We ran a 3 (age group) × 2 (gender) × 2 (drawing) ANOVA, with drawing as within-subject and age group and gender as between-subject factors. This analysis revealed only a significant effect of age, *F*(2;121) = 5.71, *p* < .01, η^2^ = .086. Posthoc Tukey tests indicated that the youngest group’s mean (23.20%) was lower than the oldest group’s mean (35.18%), whereas the middle group’s mean (31.64%) did not differ significantly from the others. The girls’ and boys’ means were 32.00% and 29.31%, respectively; however, the effects of gender, *F*(1;121) = 2.30, η^2^ = .019, *p* > .13, and drawing, *F*(1;121) = .01, η^2^ = .000, *p* > .92, were nonsignificant, as well as the interactions age group × gender, *F*(2;121) = 0.63, *p* > .53, η^2^ = .010, age group × drawing, *F*(2;121) = 0.72, *p* > .48, η^2^ = .012, gender × drawing, *F*(1;121) = 2.72, *p* > .10, η^2^ = .022, and age group × gender × drawing, *F*(2;121) = 0.09, *p* > .91, η^2^ = .001.

Table 6 reports the correlations between the main variables. All variables correlated significantly with age. The Draw-a-man correlated with both snowboarding and aikido and these correlations remained significant with age partialled out. The correlation between the snowboarding and aikido scales, *r*(125) = .44, *p* < .001, was significant and substantive also with age partialled out, *r*(124) = .39, *p* < .001. This correlation did not depend merely on drawing skills; partialling out both the Draw-a-man and age, the correlation between snowboarding and aikido drawings was still *r*(123) = .36, *p* < .001, indicating a commonality between drawing flexibility tasks.

**Table 6:** Correlations and partial correlations

	(1) D-a-M	(2) Snow	(3) Aiki	(4) Flex.	(5) Mr. C.	(6) BDS	(7) WM cap
Age	.44^***^	.33^***^	.26^**^	.35^***^	.53^***^	.59^***^	.60^***^
(1) Draw-a-man		.32^***^	.34^***^	.39^***^	.39^***^	.46^***^	.45^***^
(2) Snowboarding scale	.20^*^		.44^***^	–	.51^***^	.39^***^	.49^***^
(3) Aikido scale	.25^**^	.39^***^		–	.28^**^	.32^***^	.32^***^
(4) Average drawing flexibility	.27^**^	*–*	–		.47^***^	.42^***^	.48^***^
(5) Mr. Cucumber Test	.20^*^	.42^***^	.18^*^	.36^***^		.73^***^	–
(6) Backward Digit Span	.27^**^	.25^**^	.21^*^	.28^**^	.60^***^		–
(7) WM capacity	.26^**^	.38^***^	.21^*^	.36^***^	*–*	–	

Zero-order correlations (*d.f.* = 125) above diagonal; correlations with age partialled out (*d.f.* = 124) below diagonal in italics

Average drawing flexibility is the mean of the Snowboarding % and Aikido % scales

The correlations between variables one of which includes the other are omitted and indicated by –

^***^ *p* < .001; ^**^ *p* < .01; ^*^ *p* < .05

The hypothesis of a role of working memory capacity in drawing flexibility was supported; both the Mr. Cucumber and the Backward Digit Span, as well as their average, correlated significantly with both the snowboarding and the aikido scales, as well as with the average drawing flexibility score (i.e., the average of the snowboarding and aikido percentage scales). All nine correlations remained significant with age partialled out. In fact, drawing flexibility correlated with working memory capacity above and beyond age and the Draw-a-man score, *r*(123) = .33, *p* < .001, with both of these variables partialled out.

It is also noteworthy that the correlation between drawing flexibility and age was no longer significant when working memory capacity was partialled out, *r*(124) = .06 with snowboarding, *r*(124) = .09 with aikido, and *r*(124)=.09 with average drawing flexibility, all *p*s > .3. Furthermore, including working memory capacity as a covariate in the ANOVA reported above, the age group effect dropped to *F*(2;120) = 0.08, *p* > .91, η^2^ = .001, while the effect of working memory capacity was highly significant, *F*(1;120) = 21.82, *p* < .001, η^2^ = .154, and the other main effects and interactions remained nonsignificant. Therefore, we conclude that the linear relationship between age and drawing flexibility was fully accounted for by working memory capacity.

In a stepwise multiple regression analysis with average drawing flexibility as the dependent variable and age, working memory capacity, Draw-a-man, and gender as predictors, working memory capacity emerged as the first predictor accounting for 22.9% variance, and the Draw-a-man accounted for an additional 3.6%. Age and gender did not account for a further significant, unique portion of variance, *t*(123) = 0.46, *p* > .64, and *t*(123) = 0.15, *p* > .88, respectively. In the final model, the beta coefficients were β = .38, *p* < .001 for working memory capacity and β = .21, *p* < .02 for the Draw-a-man.

Tables 7 and 8 present the relationshipof each feature (in the snowboarding and aikido drawings, respectively) with age and working memory capacity. This is examined by comparing with *t*-tests the mean age (or the mean working memory capacity) of the participants who scored 0 or 1 on each feature. The prediction that any feature is related to age (or to working memory capacity) was tested with a Bonferroni-corrected probability of one-tailed .05/19 for snowboarding and .05/13 for aikido features. For the sake of completeness, however, Tables 7 and 8 also indicate the *t*-values significant for uncorrected two-tailed *p* < .05. As one can see, age and working memory capacity predicted scores on some, but not all features. In particular, working memory capacity was a strong predictor of nine snowboarding and two aikido features.

**Table 7:** Relationship of each feature in the snowboarding drawing with age and working memory capacity

	Age			Working memory capacity		
	Mean (0)	Mean (1)	*t*	Mean (0)	Mean (1)	*t*
Tilted board	98.4	109.8	3.63*°	3.06	3.93	5.80*°
Tilted body axis	102.0	110.8	2.45*	3.34	3.99	3.65*°
Board on slope	104.3	106.5	0.40	3.49	3.83	1.22
Bent knee	101.7	112.0	2.87*°	3.33	4.06	4.11*°
Tilted trunk	102.6	109.5	1.88	3.40	3.86	2.53*
Tilted head	104.3	105.2	0.25	3.46	3.74	1.44
Jump	100.7	109.3	2.63*	3.27	3.85	3.59*°
Flip	104.1	108.0	0.71	3.52	3.59	0.24
Crouch	103.0	115.9	2.58*	3.48	3.85	1.43
Grab	102.8	113.2	2.39*	3.48	3.79	1.40
Legs wide apart	102.6	107.4	1.44	3.42	3.70	1.68
Feet	103.6	106.2	0.78	3.43	3.71	1.64
Angle of feet	102.0	109.7	2.22*	3.36	3.87	2.91*°
Bindings	104.0	106.8	0.66	3.45	3.87	2.01*
Arms dynamic	101.3	108.2	2.13*	3.25	3.85	3.73*°
Arms different	102.3	108.6	1.84	3.30	3.94	3.82*°
Bent elbow	102.7	115.7	2.84*°	3.42	4.17	3.25*°
Outfit	97.0	105.9	2.00*	2.78	3.67	4.08*°
Lines of movement	105.0	102.6	-0.56	3.52	3.57	0.23

Mean (0), Mean (1) = mean age, or mean working memory capacity, of the participants who scored 0 or 1 on each feature

Age expressed in months

*d.f.* = 125 for all *t*-values

* *p* < .05 two-tailed; ° *p* < .05/19 one-tailed

**Table 8:** Relationship of each feature in the aikido drawing with age and working memory capacity

	Age			Working memory capacity		
	Mean (0)	Mean (1)	*t*	Mean (0)	Mean (1)	*t*
Profile	105.1	103.2	-0.52	3.48	3.63	0.83
Tilted trunk	104.7	103.4	-0.28	3.55	3.42	-0.54
Fall	101.8	108.6	2.03*	3.41	3.70	1.73
Fall details	102.7	109.8	1.91	3.46	3.71	1.29
Step forward	103.0	109.9	1.75	3.44	3.84	2.02*
Body contact	104.0	124.0	1.86	3.51	4.17	1.18
Back foot	104.8	102.4	-0.51	3.52	3.59	0.07
Arm protrusion	100.2	107.6	2.23*	3.31	3.68	2.21*
Hand contact	105.0	101.8	-0.67	3.53	3.54	0.04
Arms dynamic	99.6	108.5	2.77*	3.16	3.82	4.15*°
Arms different	101.0	108.6	2.33*	3.35	3.74	2.42*
Outfit	98.3	107.6	2.73*°	3.06	3.76	4.19*°
Lines of movement	103.8	111.4	1.36	3.51	3.69	0.63

Mean (0), Mean (1) = mean age, or mean working memory capacity, of the participants who scored 0 or 1 on each feature

Age expressed in months

*d.f.* = 125 for all *t*-values

* *p* < .05 two-tailed; ° *p* < .05/13 one-tailed

Finally, we analyzed the frequencies of transparencies and stick drawings. Transparencies were relatively rare in the control drawings (n = 11, i.e., 8.7%), but they were more frequent in the aikido drawings (n = 36, i.e., 28.3%), and even more frequent in the snowboarding drawings (n = 56, i.e., 44.1%). The frequency of transparencies in both snowboarding and aikido drawings was significantly higher than in the control drawings (McNemar test with Yates correction, χ^2^(1) = 36.53, *p* < .001 and χ^2^(1) = 16.46, *p* < .001, respectively); it was higher in snowboarding than aikido drawings, χ^2^(1) = 8.20, *p* < .01. A variable that represents the number of drawings with transparencies produced by each child correlated negatively with age, *r*(125) = -.20, *p* < .03, which indicates a decline of transparencies with age; however, the number of transparencies was unrelated to working memory capacity, *r*(125) = .03, and to the Draw-a-man, *r*(125) = .10, both nonsignificant. It was positively correlated with the score in the aikido drawing, *r*(125) = .23, *p* < .01, indicating that the participants who made richer changes in the aikido drawing were more likely to overlook some transparency on the way, but it was uncorrelated with the snowboarding drawing score, *r*(125) = .06.

Stick figure features were quite rare in the control drawings (n = 9, i.e., 7.1%) and in the snowboarding drawings (n = 12, i.e., 9.4%) but a bit more frequent in the aikido drawings (n = 18, i.e., 14.2%). The frequency of stick figures was higher in the aikido than in the control drawings, χ^2^(1) = 5.33, *p* < .03, whereas the snowboarding drawings did not differ from either the control or the aikido drawings, χ^2^(1) = 0.31 and χ^2^(1) = 1.39, respectively. The variable representing the number of stick figures was slightly skewed and peaked, so we used Spearman correlations. It was negatively correlated with age, *r*_s_ = -.51, *p* < .001, with working memory capacity, *r*_s_ = -.40, *p* < .001, with the Draw-a-man, *r*_s_ = -.43, *p* < .001, with the aikido drawing score, *r*_s_ = -.27, *p* < .01, and with the snowboarding drawing score, *r*_s_ = -.25, *p* < .01.

## Discussion

This study explored children’s representation of human movement in two specialized motor skills, in the light of drawing flexibility research. We summarize here the results in relation to our hypotheses. The scores in the snowboarding and aikido drawings shared a significant portion of variance, thus supporting the notion of an underlying drawing flexibility dimension (hypothesis *a*). Age had a significant effect on drawing flexibility scores (hypothesis *b*), whereas (hypothesis *c*) the effect of gender was nonsignificant. Drawing flexibility was significantly related to working memory capacity (hypothesis *d*), and the relationship between age and drawing flexibility vanished when working memory capacity was controlled (hypothesis *e*). Drawing specialized skilled movements caused a sizable increase in children’s use of transparencies and, to a lesser extent, stick figure features (hypothesis *f*).

A novel finding of this study was the correlation between the scores in two drawing flexibility tasks that concern representing two rather different motor skills. This correlation was also significant with age and the Draw-a-man score partialled out, which suggests that there is an underlying common dimension that denotes children’s propensity and ability to modify their habitual, canonical graphic schemes (e.g., Cox, 1993; Lowenfeld & Brittain, 1975) to represent particular meanings. This seems to add credibility to our scoring method, and we suggest that the methods we used in this study could be applied also in other research on drawing flexibility tasks, in particular to study children’s ability to represent people doing sports, dancing, or working.

Drawing flexibility tasks correlated with working memory measures, and all of these correlations were positive and significant also with age partialled out. Note that, in this context, partialling out age removes not only spurious variance (due to other age-related variables that might affect drawing flexibility), but also true variance (the maturational growth of working memory capacity). Thus, regarding a hypothesized effect of working memory capacity, partialling out age is a particularly severe test because it discards developmental differences and only retains individual differences. Still, drawing flexibility was also related to working memory capacity at the level of individual differences. In this regard, our results perfectly replicated the findings of Morra (2005). A control that was missing in Morra (2005), however, was the possibility that the correlation between working memory capacity and drawing flexibility might depend on the ability to draw the human figure. In this study, we controlled for this possibility using the Draw-a-man score and found that the correlation between drawing flexibility and working memory capacity was also significant when partialling out both age and Draw-a-man. Moreover, a regression analysis found that working memory capacity was the main predictor of drawing flexibility, and the Draw-a-man accounted for an additional but smaller portion of unique variance. Thus, in this study we found even clearer evidence than reported by Morra (2005) for the relationship between drawing flexibility and working memory.

The finding that individual differences in drawing flexibility are related to working memory capacity is not trivial. Had we not found this robust relation, that would imply that drawing flexibility only depends on other processes and variables (e.g., stimulus encoding, long-term memory, inhibition, creativity, metacognitive knowledge, or a “theory of pictures”), some of which had been proposed in the literature. We are not excluding the possibility that those processes may contribute to drawing flexibility; however, the finding of a substantive relationship between working memory and flexibility provides crucial support for Morra’s (2005) model. This model posits that drawing flexibility is a form of problem solving, i.e., it requires devising more or less effective ways for modifying a well-practiced drawing scheme. To do so, the child must have several schemes available in working memory, i.e., the (figurative) scheme that needs to be modified, one or more figurative schemes that represent relevant features of the depicted subject, and at least one operative scheme for modifying the basic, habitual graphic scheme by introducing into it (perhaps one at a time) graphic representations of the relevant features. The schemes required for graphic flexibility may have different sources of activation, including perceptual input if available, but to solve the problem of successful graphic representation it is necessary that attentional resources are allocated to fully activating a number of relevant figurative and operative schemes. A larger working memory capacity affords coordination of a larger number of relevant schemes, and thus, more complex and sophisticated solutions to the pictorial problem. For this reason, working memory capacity is a crucial variable according to Morra (2005). In this view, drawing flexibility is not just a matter of conceptual knowledge (i.e., figurative schemes), but involves an interplay between conceptual knowledge, procedural knowledge (i.e., the operative schemes that carry out graphical changes that are thought useful by the child for representing the relevant conceptual knowledge), and resources of the information processing system.

Nevertheless, younger children with smaller working memory capacity were generally able to make in the snowboarding and aikido drawings some change with respect to their control drawings (as indicated by the 23% average score of the youngest group) but, as argued in a previous section, they could find it difficult to represent events and movements while drawing their best quality human figures. This difficulty could be due to the narrow limitation of their working memory. Consider the aikido drawing in Fig. 1 and the snowboarding drawing in Fig. 2; these look quite dynamic, and for the age of their authors they are rather effective representations of the intended topics, but the representations of the human figures are quite impoverished. Both authors of these drawings had a working memory capacity that approximated two units. Following Morra (2005), we can assume that these children could make a number of “local” changes (Spensley & Taylor, 1999) by activating a figurative scheme for a relevant feature (one at a time) of the topic and an operative scheme for depicting the intended feature, but then they would not have enough capacity left for fully activating their graphic scheme for the human figure as well, which would consequently suffer a lowered quality in the drawing. In contrast, the other drawing of these two children represented the human figure somewhat better, but at the cost of less effective representation of human movement; perhaps in this case they allocated one unit of working memory capacity to activate their graphic scheme for the human figure, and thus they would not have enough capacity for also activating mental images of relevant features and operative schemes to represent them graphically.

Drawing flexibility was correlated with working memory capacity with age partialled out, but the converse was not true; with working memory capacity partialled out, drawing flexibility was uncorrelated to age. This indicates that the relationship between age and drawing flexibility is accounted for by working memory capacity. Similar results were reported by Morra (2005), where controlling for working memory capacity eliminated (in Experiments 1 and 3) or strongly reduced (in Experiment 2) the relationship between age and drawing flexibility. Thus, there is converging evidence that working memory development fuels the development of drawing flexibility. In sum, our results replicate and extend those of Morra (2005) and are broadly consistent with other research that documented a role of working memory and executive control in drawing flexibility (Blom et al., 2021; Panesi & Morra, 2016, 2022; Simpson et al., 2019). They are also broadly consistent with other research showing a major role of working memory capacity growth in cognitive development (for reviews, see Morra et al., 2008; Pascual-Leone & Johnson, 2021).

We also analyzed in detail two sets of features that children used, more or less frequently, to represent the intended content. Unsurprisingly, the scores in both snowboarding and aikido drawings increased with age; however, not all features showed the same age pattern. In the snowboarding drawing, a tilted board, tilted body axis, bent knee, and bent elbow most clearly increased with age, whereas representation of the outfit was already frequent in the youngest group, although it still increased with age. Furthermore, jump, legs wide apart, salient feet, dynamic arms, and arms in different positions were rather frequently represented (by more than one-third of participants); jump and dynamic arms also tended to increase with age. The relationship of the aikido drawing with age, albeit significant, was less strong; representation of outfit was the most frequent feature and the only one that clearly increased with age. Also, representations of fall, arm protrusion, dynamic arms, and arms in different positions were rather frequent, and all of these tended to increase with age.

These patterns of different frequency of features invite a reflection on the cognitive processes involved in our tasks. Actually, participants had first to watch the videos and encode their content, which requires attention, working memory, and long-term memory encoding; then, they had to draw what they had seen, which involves retrieving the encoded information, coordinating it in working memory, and using it to solve the problem of pictorial representation. Our technique enables us to detect age-related and working memory-related differences, but does not enable us to distinguish to what extent those differences arose at stimulus encoding or solving pictorial problems; it is conceivable that both types of processes are sensitive to developmental differences. Some details are instructive, however. For instance, in the aikido drawing, arm movements were represented quite often; hand contact was depicted only by one-seventh of participants, and body contact was quite rare, although it is very important in most of the aikido techniques shown in the videos. As aikido experts know, the most important details in a technique are often the least salient visually, and instructors need to cope with the beginners’ tendency to focus instead on what is perceptually salient. In this study, all participants were inexperienced in snowboarding and aikido, which was a way to control for the experience variable; however, it is likely that participants with experience would encode the stimulus videos differently and more deeply. It would be interesting, in future research, to introduce manipulations that enable distinguishing effects at encoding and problem solving.

Examining the relationship between working memory capacity and each specific feature of the drawings, we also note that among the snowboarding features most related to working memory capacity, several concerned angles (the tilted board, tilted body axis, etc.); we suggest that planning a drawing of a meaningful angle requires taking into account several parameters, both geometrical (such as the orientation of the frame of reference) and physical (e.g., the effect of gravity, or the way certain body movements can compensate centrifugal force). A larger working memory could facilitate participants in considering these parameters while planning their drawings. This seems akin to Piaget’s water-level task, in which to draw water as horizontal one needs to draw it at an angle with the tilted bottle sides. Neo-Piagetian research has documented the role of working memory capacity (specifically, measures of M capacity as defined in that theoretical context) in focusing attention on the relevant parameters and thus drawing the water line correctly (for an explicit model and relevant data, see Pascual-Leone & Morra, 1991; Morra, 2008b). In addition, the arms position features, in both snowboarding and aikido drawings, were highly related to working memory capacity. Representing, as “local” changes, dynamic and different arm positions was not impossible for children with a narrow working memory capacity; however, a larger capacity presumably helped children understand the effect of arm movements on one’s body balance in the case of snowboarding, or on the opponent’s body imbalance in the case of aikido, and thus recognize these as features that need be represented. Finally, in both drawings, outfit representation was related to working memory capacity. We do not see any particular reason why this should be, but probably the explanation is simply that the persons’ outfits were highly salient in all videos and thus outfit representations were present in a majority of drawings; it was especially children with a narrower working memory capacity who disregarded this detail in drawing.

The presence of transparencies was more likely in the aikido than in the control drawings, and in the snowboarding drawings it was even more likely. In the snowboarding drawings, most transparencies regarded either the board as visible through the person’s legs or some part of the person visible through the outfit. Possibly, if a child started drawing the board before drawing the snowboarder, a transparency would be inevitable; similarly, drawing the person and then adding, for instance, a helmet would also necessarily produce a “transparent” helmet through which the head contour is visible. The high rate of transparencies in the snowboarding drawing suggests that planning was often careless, e.g., not considering that to avoid transparencies the person should be drawn before the partially occluded board, or the helmet before the partially occluded head. In the aikido drawings, most cases regarded “transparent” arms, i.e., one arm visible through the other or the body visible through the arms. Also in this case, we think that the children who drew one or two “transparent” arms had not planned their posture well in advance, but rather modified it on the spot, after having drawn the body. Transparencies decreased with age, indicating that older children were better able to plan their drawing in advance, thus refraining from producing transparencies (for a detailed account of transparency in children’s drawings, see also Morra, 2008c). However, a positive correlation of transparencies with the aikido drawing score suggests that the participants who made more changes to the human figure in this drawing also had more opportunities to make some of them on the spot, thus running the risk of producing a transparency.

Only in the aikido drawing were stick figure features more likely than in the control drawing. This was akin to Kapsch and Krugel’s (2004) finding of stick figures in dance drawings. One could ask whether this was a regression to an earlier and more rudimentary drawing style, possibly due to difficulty of the task, or rather, a clever representational strategy similar to trends toward abstraction in modern art (e.g., Pablo Picasso notoriously claimed that it took him only a few years to learn to paint like Raphael but a lifetime to learn to paint like a child, and Picasso’s (1945) lithographs of a bull are an excellent example of abstraction toward simplified line drawings). In our study, the presence of stick figure features showed a strong negative correlation with age, and it also correlated negatively with working memory, Draw-a-man, and drawing flexibility. The finding that it was especially younger and/or less skilled children who tended to produce stick figure features seems consistent with the regression interpretation. This seems an example of what we noted above, i.e., that for children with a narrow working memory processing the information needed for representing movement could exhaust their available resources, which would not suffice to also keep fully activated the scheme of the human figure, thus resulting in impoverished representation (see the aikido drawing in Fig. 1 and the snowboarding drawing in Fig. 2). In contrast, the drawings in Fig. 5 illustrate how a child with a larger working memory could plan carefully how to draw modified human figures, without committing transparencies or regressing to stick figures.

One of our hypotheses, i.e., the existence of gender differences, was not supported by the results; although the girls’ mean score was slightly higher, the difference did not reach significance. Given a relatively large sample size and the small effect size, we do not think that this was due to lack of power. However, this points to a possible limitation of our study, i.e., having considered only two activities (snowboarding and aikido). Future research could also consider other activities, as well as having participants rate their interest in them, because gender differences in this domain of drawing might also be related to gender differences in the interest in the depicted topics.

Another limitation of this study was having only participants who were totally inexperienced in both snowboarding and aikido. This was deemed appropriate as a way to control for experience; however, it prevented us from studying the effect of this variable. As suggested above, experienced participants would probably encode the stimulus materials more accurately and deeply. Future research could consider some complex motor skill for which reasonably large samples of expert and naïve participants could be compared. Working memory growth and learning from specific experience are different mechanisms, however; therefore, we speculate that they could have distinguishable and additive effects. Experience could especially affect encoding, while working memory and executive control might especially constrain planning and execution of the drawing. These hypotheses could be tested in future research. Moreover, future research could investigate a possible role of experience in activities that bear some similarity to the target ones. For example, we did not ask participants if they had any experience in activities akin to snowboarding (e.g., skiing, skateboarding) or to aikido (e.g., judo, jujutsu); we acknowledge this as a limitation of our study.

Another, more important limitation of this study was due to practical reasons, because our research was carried out during the COVID-19 pandemic, so it was wiser to involve each participant in only one session, for as short a time as possible. This prevented us from including in this study a larger number of tasks and measures, which would have enabled comparison with other theoretical accounts of drawing flexibility – but would have needed at least two sessions for each participant. As the pandemic now fortunately seems to be over, future research can be more systematic in this regard.

In particular, future research could consider the possible role of inhibitory control and executive functions in these and other similar tasks; research with younger children (Panesi & Morra, 2016; Simpson et al., 2019) found a role of executive function in preschoolers’ drawing flexibility, possibly with a mediation of fine motor control (Simpson et al., 2019), and it would be interesting to examine whether this is also the case with schoolchildren.

In conclusion, this study has yielded an advance in knowledge on how children represent two complex motor skills such as snowboarding and aikido, on drawing flexibility in general, and on how working memory is involved in drawing flexibility. Moreover, we think that the method used in this study can be adopted in future research to study children’s representation of other complex human movements and skills.

## Supplementary Information

Below is the link to the electronic supplementary material.

Supplementary file1 (PDF 222 KB)

Video 1 (MOV 108 MB)

Video 2 (MOV 20.0 MB)

Video 3 (MP4 21.3 MB)

Video 4 (MOV 27.1 MB)

Video 5 (MOV 12.2 MB)

Supplementary file2 (PNG 101 KB)

Supplementary file3 (PNG 74.7 KB)

Supplementary file4 (PNG 218 KB)

Supplementary file5 (PNG 118 KB)

Supplementary file6 (PNG 257 KB)

## Data Availability

Not applicable.

## Footnotes

1 Our specific choice of snowboarding and aikido, motivated in the first place by the need to control for the participants’ experience, was also prompted by the authors’ own expertise. When this research started, one of the authors of this article was a snowboard instructor appointed by F.I.S.I. (the Italian Federation of Winter Sports), affiliated to C.O.N.I. (the Italian National Olympic Committee); another author was an aikido instructor, ranking as *sandan* in Kokusai Aikido Kenshukai Kobayashi Hirokazu Ryu, affiliated to Dai Nippon Butoku Kai.

2 The terms *seme* and *uke* refer, respectively, to who performs a martial technique and who receives it.
