# Supplementary material for: Children’s representation of specialized skilled movements: The cases of snowboarding and aikido
Source: Mem Cognit. 2024 Feb 9;53(1):54–75. doi: 10.3758/s13421-024-01522-x (PMC11779761; doi:10.3758/s13421-024-01522-x)
Supplement: Supplementary file 1 — (PDF 222 KB) [file 13421_2024_1522_MOESM1_ESM.pdf]

SUPPLEMENTARY MATERIAL 1

Table S1. **Examples of scoring** referring to the drawings presented in Figures 1-6.

	Child 1	Child 2	Child 3	Child 4	Child 5	Child 6
Snowboard drawing						
Tilted board	1	1	0	1	1	1
Tilted body axis	0	1	0	1	1	0
Board on slope	0	0	0	0	0	0
Bent knee	0	0	1	1	1	0
Tilted trunk	0	1	0	1	1	0
Tilted head	1	1	0	1	1	0
Jump	0	0	1	1	1	0
Flip	0	0	0	0	1	0
Crouch	0	0	0	0	0	0
Grab	0	0	0	0	1	0
Legs wide apart	0	0	0	1	0	0
Feet	0	1	0	0	0	0
Angle of feet	0	0	0	0	0	1
Bindings	0	1	0	0	0	0
Arms dynamic	0	0	1	1	1	0
Arms different	0	0	1	1	1	0
Bent elbow	0	0	0	1	1	0
Outfit	0	0	1	1	1	1
Lines of movement	0	0	0	0	0	0
Total snowboard	***2***	***6***	***5***	***11***	***12***	***3***
Aikido drawing						
Profile	0	0	1	1	1	0
Tilted trunk	1	0	0	0	0	0
Fall	1	0	1	1	0	1
Fall details	1	0	1	1	0	1
Step forward	1	0	0	0	1	0
Body contact	0	0	0	0	0	0
Back foot	0	0	1	0	0	0
Arm protrusion	1	1	0	1	1	1
Hand contact	0	1	0	0	1	0
Arms dynamic	0	0	0	1	1	1
Arms different	1	1	1	1	1	1
Outfit	0	1	1	1	1	0
Lines of movement	0	0	0	0	0	1
Total aikido	***6***	***4***	***6***	***7***	***7***	***6***
Average drawing flexibility (snowboard% + aikido%) / 2	**28,34**	**31,17**	**36,23**	**55,87**	**58,50**	**30,97**

Goodenough's Draw-a-man test

Instructions

"I would like you to make a picture of a man on this sheet of paper. Make the very best picture that you can. Take your time and work very carefully. Try very hard and do the best picture you can make."

Scoring checklist

1. Head
2. Legs (two; one or two if profile)
3. Arms (two; one or two if profile)
4. Trunk
5. Trunk length > breadth
6. Shoulders
7. Arms and legs attached to trunk
8. Arms and legs attached to trunk at correct positions
9. Neck
10. Neck outline continuous with head or trunk
11. Eyes (one or two)
12. Nose
13. Mouth
14. Nose and mouth in two dimensions; two lips
15. Nostrils
16. Hair
17. Hair on more than head circumference, better than scribble, and not transparent
18. Clothing
19. At least two non-transparent pieces of clothing
20. Entire drawing without transparencies; sleeves and trousers are depicted
21. At least four pieces of clothing clearly indicated
22. Complete costume with no incongruity
23. Fingers
24. Fingers: correct number

25. Fingers in two dimensions and with correct proportions
26. Thumb clearly different from other fingers
27. Hand (distinct from both arm and fingers)
28. Arm joint (elbow, shoulder, or both)
29. Leg joint (hip, knee, or both)
30. Head no less than $\frac{1}{10}$ and no more than $\frac{1}{2}$ of trunk
31. Arms: correct proportion
32. Legs: correct proportion
33. Feet in two dimensions and with correct proportion
34. Both arms and legs in two dimensions
35. Heel
36. Motor coordination: firm lines without marked tendency to cross, gap, or overlap
37. Motor coordination: all lines firm
38. Head outline better controlled than simple round shape and without unwanted irregularities
39. Trunk outline (same criteria as 38)
40. Arms and legs without irregularities, in two dimensions, and not narrowing at the junction with trunk
41. Symmetrical face features
42. Ears (two; one if profile)
43. Ears: position and proportion
44. Eyebrows or eyelashes
45. Eye detail: pupil
46. Eye detail: proportion (length > height)
47. Eye detail: glance in profile
48. Chin and forehead
49. Projection of chin
50. Profile (head, trunk, and feet)
51. Entire figure in profile without errors or transparencies

(for further explanation and examples, see Goodenough, 1926/1977).
