# Supplementary figures and images for: Children’s representation of specialized skilled movements: The cases of snowboarding and aikido
Source: Mem Cognit. 2024 Feb 9;53(1):54–75. doi: 10.3758/s13421-024-01522-x (PMC11779761; doi:10.3758/s13421-024-01522-x)

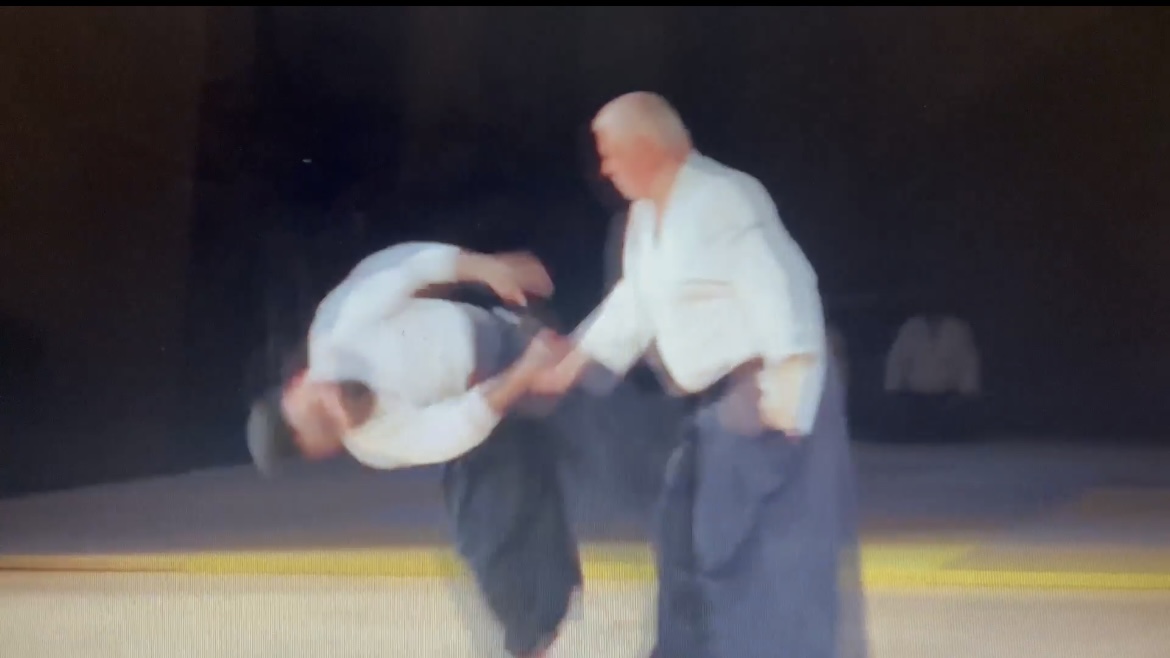

Supplement: Supplementary file 7 — (PNG 101 KB) [file 13421_2024_1522_MOESM7_ESM.png]

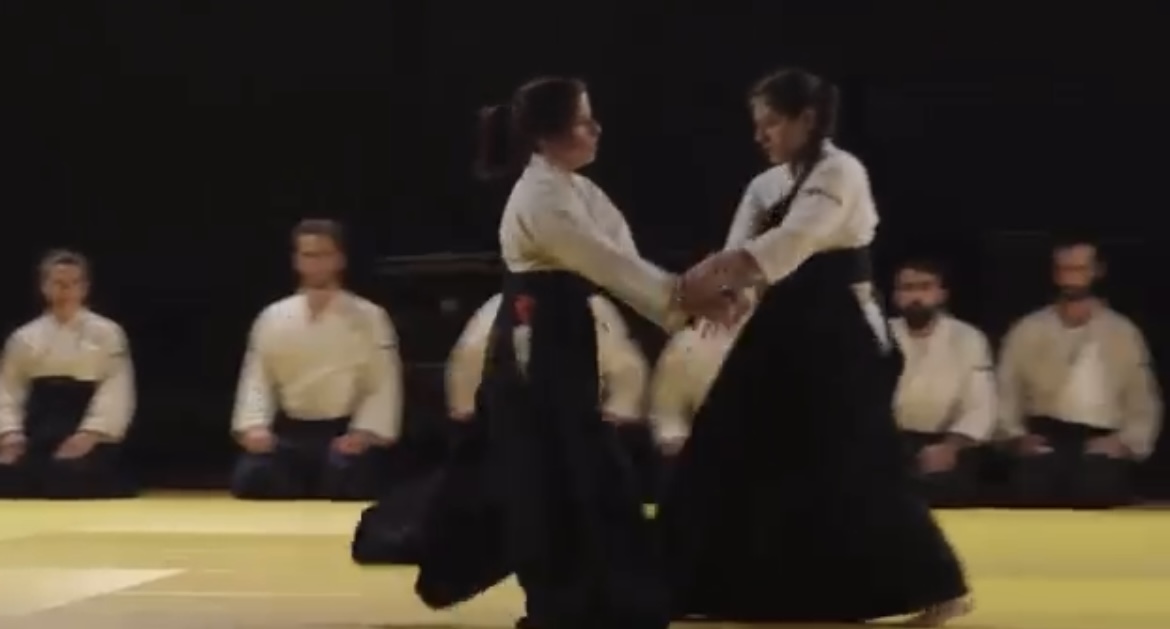

Supplement: Supplementary file 8 — (PNG 74.7 KB) [file 13421_2024_1522_MOESM8_ESM.png]

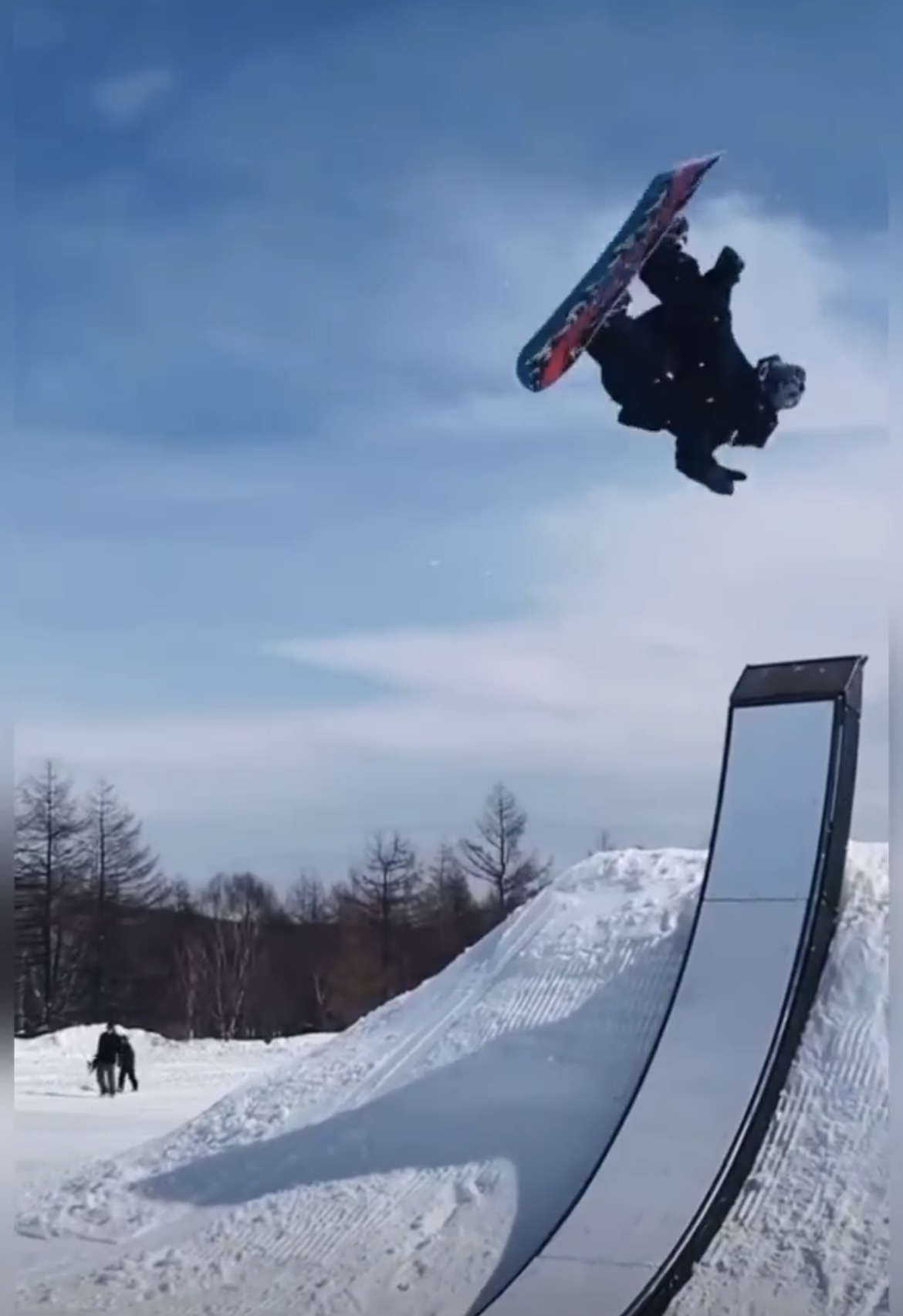

Supplement: Supplementary file 9 — (PNG 218 KB) [file 13421_2024_1522_MOESM9_ESM.png]

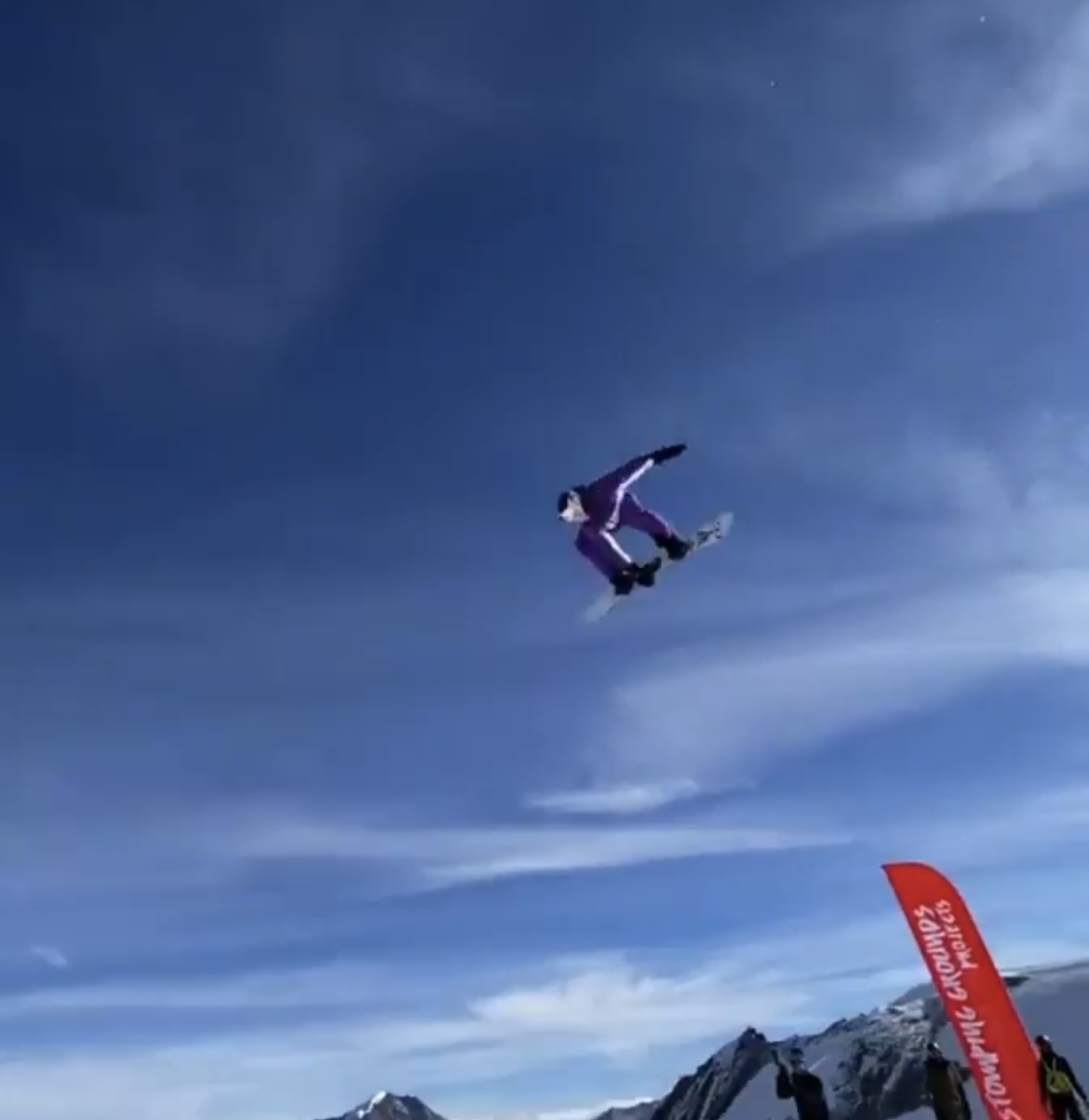

Supplement: Supplementary file 10 — (PNG 118 KB) [file 13421_2024_1522_MOESM10_ESM.png]

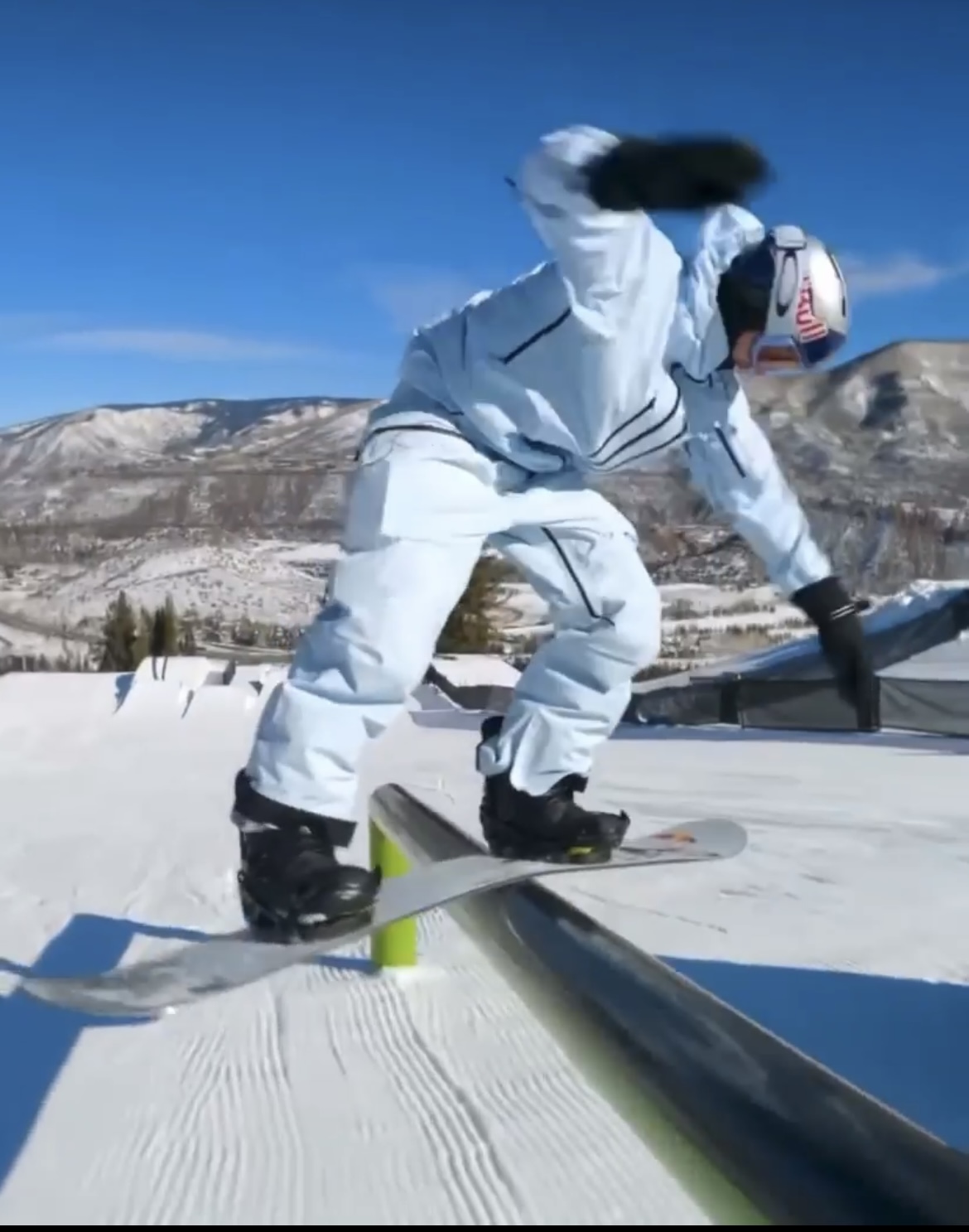

Supplement: Supplementary file 11 — (PNG 257 KB) [file 13421_2024_1522_MOESM11_ESM.png]
